# Differential expression of lysosome-associated protein transmembrane-4 beta (LAPTM4B) in granulosa cells of ovarian follicles and in other bovine tissues

**DOI:** 10.1186/s13048-015-0148-0

**Published:** 2015-03-26

**Authors:** Kalidou Ndiaye, Paul D Carrière, Jean Sirois, David W Silversides, Jacques G Lussier

**Affiliations:** Centre de recherche en reproduction animale, Département de biomédecine vétérinaire, Faculté de médecine vétérinaire, Université de Montréal, P.O. Box 5000, St-Hyacinthe, Québec J2S 7C6 Canada

**Keywords:** Ovary, Follicle, Granulosa cells, LAPTM4B, Bovine, Ubiquitination

## Abstract

**Background:**

LAPTM4B is a member of the lysosome-associated transmembrane protein superfamily that is differentially expressed in normal human tissues and upregulated in various types of carcinomas. These proteins are thought to be involved in the regulation of cell proliferation and survival. The objective of this study was to investigate the expression of bovine LAPTM4B during ovarian follicular development and in various bovine tissues.

**Methods and results:**

Northern blot analysis revealed a 1.8 kb transcript, with highly variable steady state levels among tissues. RT-PCR analysis showed that LAPTM4B mRNA transcripts were low in granulosa cells of small antral follicles, increased in large dominant follicles, and decreased in ovulatory follicles following injection of human chorionic gonadotropin (hCG; P < 0.003). Ovulatory follicles collected at various times after hCG injection revealed a significant reduction of LAPTM4B mRNA starting at 18 h post-hCG (P < 0.029). Immunobloting analysis using antibodies generated against bovine LAPTM4B recognized proteins of 26.3 and 31.5 kDa in granulosa cells of developing follicles and corpus luteum. Further analyses of affinity-purified His-tag LAPTM4B overexpressed in HEK cells showed that the 31.5 kDa protein represented the ubiquinated isoform of the 26.3 kDa native protein. The 26.3 kDa protein was differentially expressed showing highest amounts in dominant follicles and lowest amounts in ovulatory follicles 24 h post-hCG. Immunohistochemical analyses of LAPTM4B showed marked heterogeneity of labeling signal among tissues, with LAPTM4B mainly localized to perinuclear vesicles, in keeping with its putative lysosomal membrane localization.

**Conclusion:**

This study reports for the first time that bovine LAPTM4B in granulosa cells is present in both unubiquinated and ubiquinated forms, and is differentially expressed in developing ovarian follicles, suggesting a possible role in terminal follicular growth.

## Background

The lysosome-associated protein transmembrane 4 beta (LAPTM4B) belongs to the LAPTM gene family along with LAPTM4A and LAPTM5. These proteins are classified according to the number of their transmembrane domains [1,2] and are thought to be involved in the regulation of cell proliferation and survival [3,4]. The members of the LAPTM family share similarities to lysosomal proteins since they are membrane proteins that contain multiple tyrosine residues. These tyrosine-based motifs function as addressing signals, targeting the proteins toward lysosomes or late endosomes [3,5-7]. Specifically, LAPTM4B was localized to lysosomes as well as to the plasma membrane and internal organelles such as the Golgi apparatus and endosomes [8]. LAPTM4B mRNA was originally reported to be overexpressed in human hepatocellular carcinoma [9] and widely expressed in other normal human tissues, including total human ovarian extract [10]. Furthermore, its mRNA level was increased in various types of carcinomas including breast, ovarian, uterine, lung and gastric cancers [11-14]. On a functional basis, LAPTM4B promotes autophagy, a cell survival mechanism mediated by lysosomes that renders tumor cells resistant to metabolic and genotoxic stress, which promotes a faster tumor growth rate [15]. Moreover, knockdown of LAPTM4B mRNA using siRNA resulted in increased sensitivity of tumor cells to anthracyclines, an intercaling agent and a free radical inducer. Further analyses indicated that LAPTM4B overexpression resulted in specific sequestration of anthracycline and delayed the entry of anthracycline into the nucleus [15].

LAPTM4B has been studied mainly in human and specifically in regard to its association with various carcinomas, as referenced above. In the present study, we investigated the expression, localization, and regulation of the LAPTM4B mRNA and protein in the bovine species specifically during the final stages of ovarian follicular development. In a preliminary expression study, we observed that LAPTM4B mRNA was strongly expressed in granulosa cells of dominant or preovulatory follicles. We also extended the analysis to other bovine tissues to allow for comparisons since no data is available in the bovine species.

The cyclic ovarian activity results in profound modifications that require spatio-temporal coordination of proliferation, apoptosis and differentiation of various cell types within the follicles resulting from changes in gene expression. The growth of antral follicles is mainly under the influence of the follicle-stimulating hormone (FSH) [16]. During follicular selection and dominance, granulosa cells acquire luteinizing hormone receptors (LHr) that allows the transfer of follicle dependency from FSH to LH and the increase in synthesis of oestradiol-17β [17,18]. The preovulatory surge of LH from the anterior pituitary gland results in ovulation and the release of the oocyte, and the remaining granulosa and theca cells of the follicle wall differentiate into luteal cells to form the corpus luteum and produce progesterone [19]. Granulosa cells thus play a critical role in reproductive functions as they contribute to steroid hormone synthesis [20], oocyte maturation [21], and corpus luteum formation after ovulation [22]. The transcription of genes in granulosa cells that control the growth of a bovine dominant or preovulatory follicle is rapidly downregulated or silenced as a result of LH-mediated increases in intracellular signaling [23]. Conversely, LH upregulates or induces the expression of genes involved in ovulation and luteinization [24-26]. These studies demonstrate the critical importance of gene expression and regulation studies during the final stages of follicular development and ovulation. In this study, we report the differential regulation and localization of LAPTM4B in granulosa cells during the periovulatory period as well as in other bovine tissues that lead to a better understanding of its physiological function.

## Methods

### Experimental animal models

The regulation of LAPTM4B expression during follicular development and ovulation was studied using *in vivo* models as previously characterized [23]. Estrous cycles of normal cycling crossbred heifers were synchronized with one injection of PGF_2α_ (25 mg, im; Lutalyse, Upjohn, Kalamazoo, MI) given in the presence of a corpus luteum, and ovarian follicular development was monitored by daily transrectal ultrasonography. Following estrous synchronization, heifers were randomly assigned to the dominant follicle group (DF, n = 4), or the ovulatory hCG-induced follicle group (OF, n = 4). In the DF group, the ovary bearing the DF on the morning of day 5 of the estrous cycle (day 0 = day of estrus) was obtained by ovariectomy (via colpotomy). The DF was defined as > 8 mm in diameter and growing while subordinate follicles were either static or regressing. The OF were obtained following an injection of 25 mg of PGF_2α_ (Lutalyse) on day 7 to induce luteolysis, thereby promoting the development of the DF of the first follicular wave into a preovulatory follicle. An ovulatory dose of hCG (3000 IU, iv; APL, Ayerst Lab, Montréal, QC) was injected 36 h after the induction of luteolysis, and the ovary bearing the hCG-induced OF was collected by ovariectomy at 0, 6, 12, 18, and 24 h after hCG injection (n = 2–4 cows/time point). Immediately following ovariectomy, follicles were dissected into preparations of follicular wall (theca interna with attached granulosa cells) [27] or further dissected into separate isolates of granulosa cells [23], and stored at −70°C. Additionally, GC were collected from 2 to 4 mm small follicles (SF) obtained from slaughterhouse ovaries, and a total of three pools of 20 SF was prepared. Concentrations of progesterone (P_4_), and estradiol-17β (E_2_), and their ratio (P_4_/E_2_) were validated by radioimmunoassay of follicular fluid as previously described [23]. Corpora lutea (CL) at day 5 of the estrous cycle were obtained by ovariectomy and were dissected from the ovarian stroma, frozen in liquid nitrogen, and then stored at −70°C. The Animal Ethics Committee of the Faculty of Veterinary Medicine of the University of Montreal approved all animal procedures.

### Cloning of bovine LAPTM4B

The LAPTM4B cDNA was cloned from a bovine cDNA library prepared with polyA^+^ mRNA isolated from GC of dominant follicles at day 5 of the estrous cycle as described above. The cDNA library was constructed in lambda Zap Express vector (Stratagene, La Jolla, CA) by unidirectional cloning of cDNAs as previously described [28]. Following *in vivo* excision of pBluescript phagemids containing the cloned cDNA insert with the Ex-Assist/XLOLR system (Stratagene), single bacterial colonies were randomly picked and their phagemid content were purified by mini-prep (Qiagen, Mississauga, ON). The LAPTM4B cDNA was entirely characterized by sequencing on an ABI Prism 310 (Applied BioSystem).

The 5′-end of the bovine LAPTM4B was verified by screening a genomic DNA library to eventually yield all the 5′-untranslated region and promoter sequences. The genomic DNA library was prepared in Lambda phages (BD Biosciences Clontech) and 1×10^7^ pfu was screened using the bovine LAPTM4B cDNA (GenBank NM_205802) as a P^32^-radiolabelled probe. Hybridized clones were analyzed by PCR using specific probes designed in the 5′-UTR of the LAPTM4B cDNA (forward: GCGAGCTCTTCGCGGGGAGAG; reverse: CAAGTACCAGACGCCGAGCAG). A second round of screening was performed to isolate a positive clone. Recombinant DNA was purified as described [29], cloned into the pDrive vector (Qiagen Cloning kit) following *Bam*H1 digestion, and characterized by sequencing.

### mRNA expression analysis

RNA was isolated from various bovine tissues obtained from slaughterhouse by extraction in lysis buffer (4 M guanidium isothiocyanate, 0.5% Na-N-laurylsarcosine, 25 mM Na-citrate, pH 7), sedimentation, and centrifugation on a cesium chloride cushion as previously described [28]. The concentration of total RNA was quantified by measurement of optical density at 260 nm, and quality was evaluated by visualizing the 28S and 18S ribosomal bands following electrophoretic separation on 0.66 M formaldehyde denaturing 1% agarose gel with ethidium bromide [29]. Expression of LAPTM4B mRNA in various tissues was compared by Northern analysis. Total RNA (20 μg/tissue) was size-fractionned on a 0.66 M formaldehyde 1% agarose gel, transfered by capillarity onto a nylon membrane (Hybond-N; GE Healthcare Life Sciences), and UV-treated (150 mJ) as previously described [28]. The amount of ribosomal RNA (18S) was estimated after methylene blue staining and the image was digitized (FotoDyne Inc., Hartland, WI) and analyzed using the NIH Image software. The bovine LAPTM4B cDNA was used to generate a radioactive probe incorporating [α^32^P]-dCTP (NEN Life Sciences, Boston, MA) that was subsequently used to hybridize Northern blots as described [28]. The film images were digitized and the intensity of bands expressed as ratios of 18S ribosomal RNA.

Expression and regulation of *LAPTM4B* mRNA during follicular development and following hCG injection was analyzed by semi-quantitative RT-PCR. Total RNA was extracted from bovine GC collected from follicles at different developmental stages (SF, DF, OF) and CL, and from follicular wall (granulosa and theca cells) collected at 0, 6, 12, 18, and 24 hours after an injection of hCG. The sample at 0 hour was represented by day 7 dominant follicle (DF). Specific *LAPTM4B* PCR primers were used and the number of cycles was limited and optimized for analysis of *LAPTM4B* mRNA expression. PCR reaction products were separated on a 2% TAE-agarose gel with ethidium bromide, visualized by UV light, digitized and analyzed by densitometry using ImageQuant software (GE Healthcare Life Sciences). *GAPDH* was used as a control gene, and specific signals of *LAPTM4B* were normalized with corresponding *GAPDH* signals.

### Production of polyclonal anti-bovine LAPTM4B antibodies

A fragment corresponding to amino acids Lys^169^ to Ala^225^ (LAPTM4B; Molecular weight = 7.5 kDa) located at the carboxy-terminal end of the bovine LAPTM4B was used to generate a glutathione S-transferase fusion protein (GST Gene Fusion System; GE Healthcare Life Sciences) to produce specific polyclonal antibodies. To increase the molecular weight of LAPTM4B recombinant protein fragment and thus facilitate down-stream purification procedures, a DNA construct that included a tandem repeat of Lys^169^ to Ala^225^ (LA)_2_-LAPTM4B fragments cloned at the carboxy-terminal end of the GST was generated. A set of forward (5′-AAGGGTTACTTGATTAGCTGTGTTTGG-3′) and reverse (5′-GGCAGACACGTACGGGGGC-3′) primers that incorporated *Bam*HI (forward primer) and *Eco*RI (reverse primer) restriction sites were designed from the LAPTM4B cDNA to generate the first LAPTM4B fragment. The same set of primers that incorporated an *Eco*RI (forward primer) and *Sal*I (reverse primer) were used to generate the second LAPTM4B fragment. The LAPTM4B fragments were amplified by PCR using the Expand High Fidelity polymerase (Roche Molecular Biochemicals) according to the manufacturer’s protocol. The fragments were isolated after electrophoresis, digested with either *Bam*HI and *Eco*RI (for the first fragment), or *Eco*RI and *Sal*I (for the second fragment). The first fragment was subcloned into the pGEX-2 T vector (GE Healthcare Life Sciences) in frame with the GST coding region, as described previously [28]. The second fragment was then subcloned down-stream of the GST-LAPTM4B first fragment, and the resulting plasmid sequenced to confirm its integrity. Protease-deficient *E. coli* BL-21 (GE Healthcare Life Sciences) were transformed with the (LA)_2_-LAPTM4B/pGEX-2 T construct, and expression of recombinant (LA)_2_-LAPTM4B/GST fusion protein was induced with 0.1 mM isopropyl-1-thio-β-D-galactopyranoside (IPTG) for 6 hours. Proteins from bacterial extracts were obtained after sonication as previously described [28]. The (LA)_2_-LAPTM4B/GST-fusion protein was purified by affinity on glutathione-Sepharose beads (GE Healthcare Life Sciences), and digested with thrombin (10units/1 mg of fusion protein) to release the tandem (LA)_2_-LAPTM4B fragment. Proteins were resolved by one-dimensional SDS-PAGE, transferred onto nitrocellulose membrane (0.45 μm Hybond C; GE Healthcare Life Sciences), and stained with Ponceau S red. The tandem (LA)_2_-LAPTM4B band (MW = 15.3 kDa) was cut and used to immunize a rabbit as previously described [28]. Preceeding immunization, the identity of the purified (LA)_2_-LAPTM4B fragment was verified by liquid chromatography-tandem mass spectrometry (Eastern Quebec Proteomics Center, Laval University, Quebec, Canada). Polyclonal antibodies against bovine LAPTM4B were validated by immunoblotting using the GST-affinity purified recombinant (LA)_2_-LAPTM4B.

### Cell extracts and immunoblotting analysis

Granulosa cells and CL were obtained as described above. They were homogenized in M-PER buffer (Pierce, Rockford, IL, USA) supplemented with complete protease inhibitors (Roche Diagnostics, Laval, QC, Canada) as described by the manufacter’s protocol, and centrifuged at 16,000 × *g* for 10 min at 4°C. The recovered supernatant was stored at −70°C until electrophoretic analyses were performed. Protein concentrations were determined according to Bradford method [30] (Bio-Rad Protein Assay, Bio-Rad Lab, Mississauga, ON, Canada). Immunoblotting was performed as described previously [28]. Samples (50 μg of proteins) were resolved by one-dimensional denaturing Novex Tris-glycine gels (Invitrogen, Burlington, ON, Canada) and electrophoretically transferred to polyvinylidene difluoride membranes (PVDF; GE Healthcare Life Sciences). Membranes were incubated with the polyclonal anti-bovine LAPTM4B antibody (1:2000), and immunoreactive proteins were visualized by incubation with horseradish peroxidase-linked donkey anti-rabbit secondary antibody (1:20 000 dilution) and the enhanced chemiluminescence system, ECL plus (GE Healthcare Life Sciences) according to the manufacturer’s protocol followed by revelation using the ChemiDoc XRS+ system (Bio-Rad).

### Overexpression of bovine LAPTM4B in mammalian cells

To confirm the detection of the two forms of LAPTM4B protein with our antibodies, overexpression experiments in mammalian cells were performed. The LAPTM4B open reading frame was amplified by PCR using the Expand High Fidelity polymerase (Roche Molecular Biochemicals) with specific LAPTM4B primers. The PCR fragment was purified and cloned into pQE-TriSystem His-Strep2 (Qiagen). The final construct pQE2-LAPTM4B was used to transfect HEK cells using the CalPhos mammalian transfection kit (Clontech Laboratories, Inc.) according to the manufacturer’s protocol. Seventy-two hours post-transfection, cells were harvested and protein extraction as well as immunoblotting was performed as described above using the anti-LAPTM4B antibody generated from the present study and a commercial anti-LAPTM4B antibody (Abcam Inc.; cat. # ab82810, at a concentration of 1 μg/ml). To verify for ubiquitination of the LAPTM4B protein, immunoblotting was also performed using a monoclonal anti-ubiquitin antibody (Sigma U0508 at 1:2,500 dilution). Immunoreactive proteins were visualized by incubation with horseradish peroxidase-linked sheep anti-mouse secondary antibody (1:20 000 dilution) and the enhanced chemiluminescence system, ECL plus (GE Healthcare Life Sciences) according to the manufacturer’s protocol followed by revelation using the ChemiDoc XRS+ system (Bio-Rad).

### Immunohistochemistry

Immunohistochemistry was performed on PBS buffered formaline-fixed follicles and CL that were generated as described above, as well as on bovine tissues obtained from slaughterhouse. Paraffin-embedded tissues were cut at 4 μm thickness, mounted on SuperfrostPlus slides (Fischer Scientific, QC), deparaffined and rehydrated. Tissue sections were heat-treated as previously described [28], and were incubated for 14 h at 4°C with our anti-LAPTM4B antibody at a dilution of 1:500 in Tris-buffered saline (TBS; 150 mM NaCl, 0.1 M Tris pH 7.5) containing 1% bovine serum albumin, and 1% fat-free skim milk. Control tissue sections were incubated similarly with preimmune serum. The primary antibody and LAPTM4B antigen complexes were detected by incubation with a monoclonal anti-rabbit IgG conjugated with alkaline phosphatase (Sigma Chemicals) at a dilution of 1:200 for 2 h at room temperature, followed by several washes in TBS, and incubation with the NBT/BCIP alkaline phosphatase substrate (Roche Diagnostics). Sections were mounted in 5% gelatin, 27% glycerol, and 0.1% sodium azide. Photographs were taken under bright field illumination using a Nikon Eclipse E800 microscope equipped with a digital camera (Nikon DXM 1200). Digital images were processed by the Photoshop software (Adobe Systems Inc., San Jose, CA) and assembled by the Illustrator software (Adobe Systems Inc.).

### Statistical analysis

Amounts of LAPTM4B mRNA were normalized with those of the control gene GAPDH. Homogeneity of variance between groups was verified by O’Brien and Brown-Forsythe tests. Corrected values of gene specific mRNA levels were compared between follicular or CL groups by one-way ANOVA. When ANOVA indicated a significant difference (P < 0.05), the Tukey-Kramer test was used for multiple comparison of individual means among SF, DF, OF and CL, whereas the Dunnett test (P < 0.05) was used to compare different time points after hCG with 0 h as control. Data were presented as least-square means ± SEM. Statistical analyses were performed using JMP software (SAS Institute, Inc.).

## Results

### Isolation and expression analysis of bovine LAPTM4B mRNA

**Figure 1:**
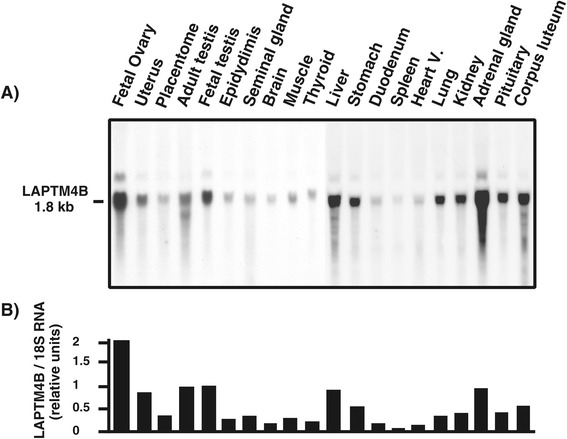
**Analysis of bovine LAPTM4B mRNA expression in bovine tissues by Northern blot.** Total RNA was extracted from various bovine tissues and samples (20 μg/well) were analyzed by Northern blot as described under Materials and Methods. **A**. The presence of a 1.8 kb transcript corresponding to LAPTM4B mRNA was detected and showed a variable pattern of expression among tissues. **B**. Comparison of relative steady state levels of bLAPTM4B mRNA expression corrected using 18S rRNA.

**Figure 2:**
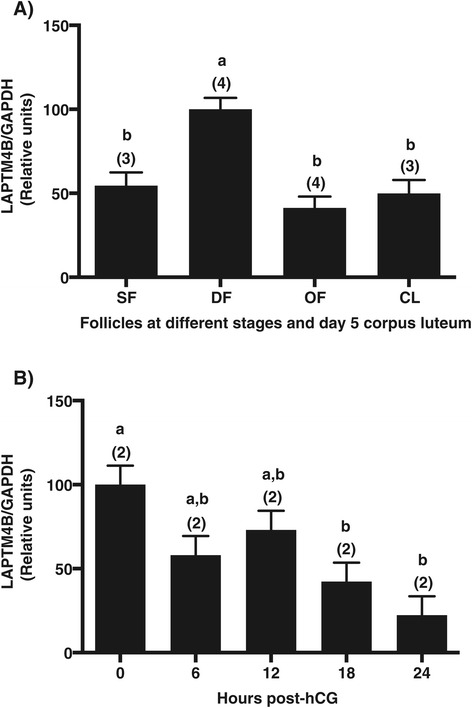
**Regulation of LAPTM4B mRNA expression during follicular development using semi-quantitative RT-PCR.** RNA samples were collected from bovine GC of 2–4 mm follicles (SF), dominant follicles at Day 5 of the estrous cycle (DF), ovulatory follicles 24 h after injection of hCG (OF), and CL at Day 5 of the estrous cycle (panel A), and from follicular walls (granulosa cells and theca interna) at different time-point: 0, 6, 12, 18 and 24 hours post-hCG (panel B); 0 h corresponds to day 7 dominant follicle. GAPDH was used as control gene, and showed no significant difference in mRNA expression levels among samples. Gene-specific signals were normalized with corresponding GAPDH signals for each sample. **A**. Expression of LAPTM4B displayed a 2-fold and 2.4-fold greater expression amounts in DF than in SF and in OF, respectively (ANOVA: *P* < 0.003). **B**. LAPTM4B expression was significantly downregulated starting at 18 hours following hCG injection compared to 0 hour (ANOVA: *P* < 0.029). Different letters denote samples that are significantly different (*P* < 0.05) when Tukey-Kramer multiple comparison test (panel A) or Dunett test (panel B) were performed. Data are presented as least-square means ± SEM, and the number of independent samples per group is indicated in parenthesis.

The of LAPTM4B cDNA was isolated from a lambda phage cDNA library constructed from bovine GC collected from dominant follicles at day 5 of the estrous cycle, and characterized by sequencing [GenBank: AF276819]. LAPTM4B expression was analyzed at the mRNA level via Northern blot analysis using several bovine tissues. A single transcript of LAPTM4B was detected at 1.8 kb, and the intensity of the signal was highly variable among tissues (Figure 1). The LAPTM4B steady state mRNA levels were strongest in fetal ovary, testis, adrenal gland, liver and uterus, moderately expressed in other tissues, and the weakest expression was observed in the spleen (Figure 1). The expression pattern of LAPTM4B during follicular development and ovulation was investigated by semi-quantitative RT-PCR using mRNA samples derived from GC at different follicular developmental stages and CL at D5 of the estrous cycle. LAPTM4B mRNA was differentially expressed with the strongest expression in DF compared to SF, OF and CL (*P* < 0.003; Figure 2A). Levels of LAPTM4B transcript increased by nearly 2-fold in GC of DF compared to SF, and declined by 2.4-fold in OF as compared to DF. Since hCG treatment decreased LAPTM4B mRNA in GC 24 h following its injection, we studied its expression in follicular walls obtained from ovulatory follicles that were isolated at different time points between 0 and 24 hours after hCG treatment (Figure 2B). A significant decline in LAPTM4B mRNA expression was observed at 18 hours following hCG when compared to 0 h (*P* < 0.029) and reached the lowest level at 24 hours.

### Production of an anti-bovine LAPTM4B antibody and immunoblotting analysis

**Figure 3:**
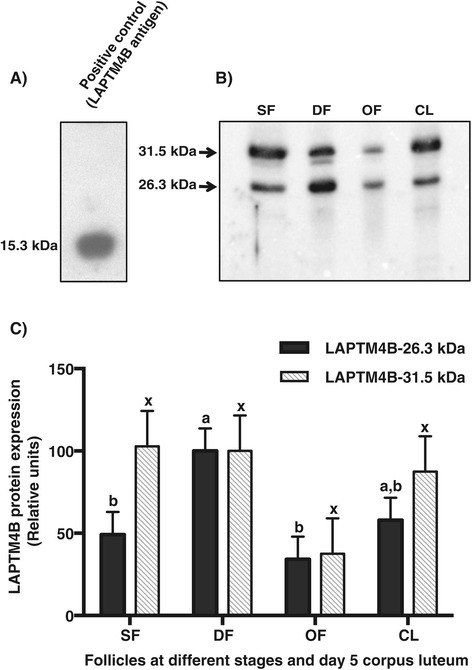
**Comparison of bovine LAPTM4B protein expression.** Total protein extracts (50 μg/well) from granulosa cells of 2–4 mm small follicles (SF), dominant follicles at Day 5 of the estrous cycle (DF), ovulatory follicles 24 h after hCG injection (OF), CL at Day 5 and the recombinant LAPTM4B fragment were size-fractionned on a 15% denaturing SDS-PAGE gel and immunoblotted using the anti-bovine (LA)_2_-LAPTM4B polyclonal antibody as described under Materials and Methods. **A**. The recombinant tandem fragment (LA)_2_-LAPTM4B was specifically recognized at 15.3 kDa. **B**. The results for the protein extracts of representative follicles and CL are shown. The native bovine LAPTM4B showed two forms migrating at 26.3 kDa and at 31.5 kDa. A stronger signal of the 26.3 kDa protein was observed in DF, and the weakest signal was observed in OF. Expression of the 31.5 kDa form was not statistically different among samples. **C**. The relative intensities of the 26.3 kDa and 31.5 kDa bands were quantified by densitometry and are represented. The results are presented as means ± SEM of two independent samples per group, and bars with different letters indicate significantly different values (*P* < 0.05).

**Figure 4:**
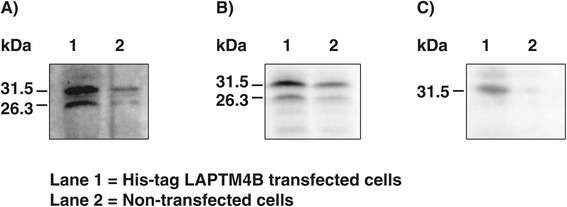
**Overexpression analysis of LAPTM4B in HEK cells.** LAPTM4B was cloned into the his-tag pQE2 vector and used to transfect HEK cells. Proteins were extracted and subjected to immunoblotting using the anti-bovine (LA)_2_-LAPTM4B polyclonal antibody (panel **A**) or a commercially available anti-LAPTM4B antibody (panel **B**). Two proteins of 26.3 and 31.5 kDa were observed with both antibodies. His-tag LAPTM4B proteins produced by HEK cells were purified by affinity chromatography and subjected to immunoblotting using a bovine ubiquitin antibody that only recognized the 31.5 kDa LAPTM4B form (panel **C**).

The recombinant bovine LAPTM4B fragment corresponding to Lys^169^- Ala^226^, with a theoritical MW of 7.5 kDa, was produced in tandem (LA)_2_-LAPTM4B and released from GST by thrombin digestion. The (LA)_2_-LAPTM4B peptide fragment migrated at 15.3 kDa on a denaturing SDS-PAGE gel and was sequenced by mass spectrometry, which confirmed its identity. The (LA)_2_-LAPTM4B was used as immunizing antigen to generate anti-bovine LAPTM4B polyclonal antibodies. Immunoblotting analyses showed that the antibody preparation recognized the affinity-purified and thrombin-cleaved (LA)_2_-LAPTM4B fragment that migrated at 15.3 kDa (Figure 3A). The antibody recognized the native LAPTM4B migrating at a MW of 26.3 kDa (Figure 3B) in total protein extracts of GC and CL, which corresponds to its theoritical MW of 25.4 kDa. A higher molecular size protein migrating at 31.5 kDa was also detected (Figure 3B). Comparison of the 26.3 kDa LAPTM4B protein expression showed greatest amounts in GC of DF whereas moderate expression was observed in CL, and significantly weaker expression was observed in OF and SF as compared to DF (*P* < 0.03; Figure 3C). The 31.5 kDa form showed no statistically significant variation among the different follicular stages and CL although its concentration was 2.6-fold weaker in OF than in DF. Overexpression experiments of the full-length LAPTM4B cDNA in HEK cells confirmed that two protein forms were produced from the single cDNA. These two forms were recognized at 26.3 and 31.5 kDa by the anti-LAPTM4B antibodies generated in this study (Figure 4A) and by the one acquired commercially (Figure 4B). The His-tag LAPTM4B protein expressed in HEK cells was affinity purified and analyzed by immunoblotting using anti-bovine ubiquitin antibodies that detected a single protein band at 31.5 kDa indicating ubiquitination of LAPTM4B (Figure 4C).

### Immunolocalization of LAPTM4B

**Figure 5:**
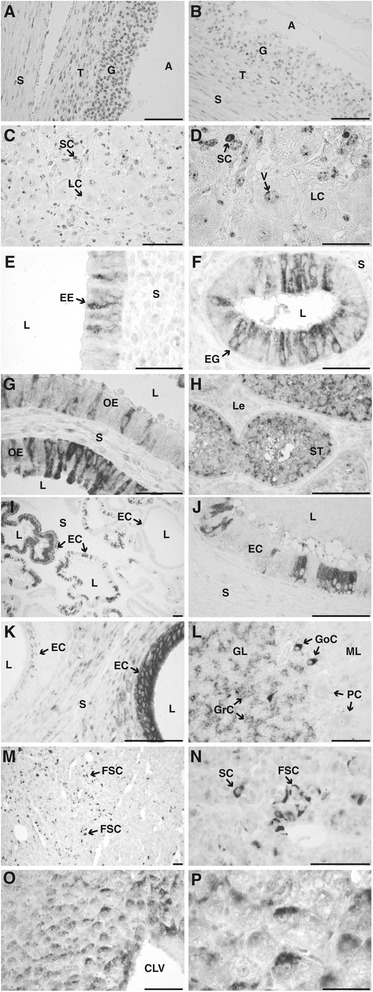
**Immunohistochemical localization of LAPTM4B in bovine tissues.** Sections of paraffin-embedded tissues were incubated with the antibody directed against the recombinant bovine LAPTM4B (1: 500 dilution) and the complex was detected with a monoclonal anti-rabbit antibody coupled to alkaline phosphatase and NBT/BCIP as substrate. No counter staining was used. *Bar* = 0.1 mm if not otherwise stated. **A**. Follicular wall of a day 5 dominant follicle; *A* antrum, *G* granulosa, *S* stroma, *T* theca interna. **B**. Follicular wall of an ovulatory follicle 24 h following hCG-injection. **C**. Corpus luteum at day 5 of the estrous cycle; *LC* large luteal cells, *SC* small luteal cells. **D**. Higher magnification of the corpus luteum section presented in C; *V* perinuclear vesicles. *Bar* = 0.05 mm. **E**, **F**. Endometrium; *EE* endometrial epithelial cells, *EG* endometrial glandular cells*, L* lumen, *S* stroma, *bar* = 0.05 mm. **G**. Oviduct; *OE* Oviductal epithelial cells, *S* stroma, *L* lumen. **H**. Prepupertal testis; *ST* Seminiferous tubule, *Le* Leydig cells. **I**. Seminal vesicle; *EC* Epithelial cells, *L* lumen, *S* stroma. **J**. Higher magnification of seminal vesicle presented in J. **K**. Tail of epididymis; *EC* epithelial cells, *S* stroma, *L* lumen. **L**. Cerebellum; *GL* granular layer, *ML* molecular layer, *GrC* granular cells, *GoC* Golgi cells, *PC* Purkinje cells. **M**. Adenohypophysis; *FSC* folliculo-stellate cells. **N**. Higher magnification of adenohypophysis presented in M. **O**. Liver; *CLV* centrolobular vein. **P**. Higher magnification of hepatocytes presented in L; *Bar* = 0.05 mm.

Immunohistochemical analyses provided evidence that LAPTM4B expression in bovine tissues is variable and specific given the pattern of expression observed even among similar cell types within a tissue. In GC, a perinuclear pattern of immunostaining was observed in all cells (Figure 5A). Dominant follicles obtained at day 5 showed a labeling signal in GC that was stronger compared to GC from follicles obtained 24 h following hCG injection (Figure 5B). Theca cells were also stained but the staining was weaker compared to GC (Figure 5A-B). For the CL, immunolabeling was variable among luteal cell types and within each cell type (Figure 5C,D). For instance, large luteal cells presented a weaker or no signal as compared to small luteal cells. Moreover, subcellular labeling was associated with perinuclear vesicles (Figure 5D). Since LAPTM4B mRNA expression in different tissues was variable among the tissues analyzed (Figure 1), we extended the immunohistochemical observations to other female and male reproductive tissues. Epithelial and glandular endometrial cells showed a variable pattern of immunostaining even among adjacent cells (Figure 5E,F). Interestingly, endometrial glandular cells that were closer to the uterine lumen showed a stronger signal compared to the endometrial cells located deeper in the gland (not shown). In oviductal epithelial cells, heterogeneity of staining for LAPTM4B was also noticeable, with weak to strong signals associated with variable localization, being either perinuclear or at the baso-lateral or at apical side of cells (Figure 5G). In the testis, the intensity of immunostaining was variable between seminiferous tubules and was associated with Sertoli cells (Figure 5H). Epithelial cells of the seminal gland showed a variable expression pattern associated with a strong to weak signal even for adjacent groups of cells (Figure 5 I,J). Epithelial cells lining the epidydimis tail ducts showed immunolabeling signal from intense to weak that varied between ducts (Figure 5K). In the granular layer of the cerebellum, granular cells were weakly stained whereas strong perinuclear labeling was associated to Golgi cells. Purkinje cells were not labeled (Figure 5L). In the adenohypophysis, strong immunolabeling was associated to the folliculo-stellate cells (Figure 5M,N). In the liver, hepatocytes located near the centrolobular vein demonstrated a stronger perinuclear signal than hepatocytes that were located eccentric to the centrolobular vein (Figure 5O,P).

## Discussion

This study is the first to report that LAPTM4B expression is differentially regulated at the mRNA and protein levels in granulosa cells at critical stages of follicular development, being stronger at the time of dominance than at recruitment (2–4 mm diameter), at the time of ovulation and in luteal cells. LAPTM4B differential expression extends to different bovine tissues. Moreover, immunohistochemical observations revealed that bovine LAPTM4B expression was highly variable among tissues and even among identical cell types of the same tissue.

The mRNA expression study of LAPTM4B in different bovine tissues revealed a single transcript of 1.8 kb, which corresponds well to the length of the cDNA characterized by sequencing from the granulosa cells cDNA library. Interestingly, two transcripts of 2.2 kb and 1.5 kb were reported in human and were named LAPTM4B variant 1 and variant 2, respectively [10,12,13,31]. The size of the human LAPTM4B variant 2 (GenBank accession number: AF527412; [10]) corresponds to the bovine LAPTM4B cDNA characterized in this study. By immunoblotting analysis of hepatocellular carcinoma and normal liver tissue, it was shown that human LAPTM4B was expressed as two isoforms that were translated from alternative ATG codons [12]. The apparent molecular weights of the isoforms were 35 and 24 kDa, corresponding to predicted 317- and 226-amino acid proteins, respectively. Expression of the 35-kDa isoform was greater in highly metastatic cell lines originating from liver, prostate, and pulmonary giant cell cancers than in syngenic low metastatic cell lines [12]. Immunoblotting analyses in the present study using antibodies raised against bovine LAPTM4B showed the presence of two proteins migrating at 26.3 kDa and 31.5 kDa, which corroborates the molecular weight of the protein observed in human [12]. However, contrary to the human LAPTM4B cDNA, the cDNA of the bovine LAPTM4B contains a single initiation codon of translation and the size of the bovine cDNA characterized herein corresponds to the size of the transcript estimated by Northern blot (1.8 kb). To further characterize and verify the 5′-UTR region of bovine LAPTM4B, a genomic library was screened to eventually yield the 5′-UTR and proximal promoter sequences. The sequencing results showed that there was a single open reading frame coding for the 26.3 kDa form. Following the synthesis of the 26.3 kDa bovine protein, post-translational modifications may result in the 31.5 kDa protein. The anti-bovine as well as commercially available anti-human LAPTM4B antibodies recognized the two protein isoforms when bovine LAPTM4B cDNA was overexpressed in HEK cells. These results suggested a specific binding to the 26.3 and 31.5 kDa proteins rather than a cross reaction with a non-related protein. The affinity purified His-tag LAPTM4B protein overexpressed in HEK cells was analyzed by immunoblotting using anti-ubiquitin antibodies and demonstrated that the 26.3 kDa protein undergoes mono-ubiquitination thereby resulting in the 31.5 kDa isoform. The difference in molecular weight between the estimated 26.3 and 31.5 kDa corresponds well to the theoritical molecular weight of ubiquitin. This result is also supported by the recent observation that ubiquitination has a role in membrane sorting of LAPTM4 proteins [8].

Comparison of mRNA steady state levels in follicles at different developmental stages has shown that LAPTM4B was expressed in GC of SF, reached strongest expression in DF and declined in OF. Similarly, immunoblotting analysis revealed a variable expression of the 26.3 kDa LAPTM4B protein that paralled the mRNA results. Amounts of LAPTM4B protein were low in SF, highest in DF and lowest in OF. The comparison of LAPTM4B expression by immunohistochemistry provided further support that it is expressed in GC of DF, and this expression is reduced in OF 24 h following hCG treatment. Immunoblotting analysis showed no significant variation in the concentration of the 31.5 kDa protein, which could be related to the stability and accumulation of the 31.5 kDa isoform in lysosomes following ubiquitination of the 26.3 kDa protein. Thus, *de novo* synthesis of LAPTM4B protein may be better reflected by the 26.3 kDa isoform, which paralleled the variations observed at the mRNA level.

The immunohistochemistry results suggest that bovine LAPTM4B has a lysosomal localization as shown by the perinuclear staining in different cell types. Located at its carboxy terminal end, LAPTM4B contains a lysosomal targeting motif and was also shown to co-immunoprecipitate with ubiquitin ligase Nedd4 [3,8]. Ubiquitination of LAPTM4B by Nedd4 may contributes to its targeting towards late endosomes and lysosomes [8,32]. These observations correlate well with a lysosomal localization as previously observed for human LAPTM4B [8] and for LAPTM4A [4,33]. Interestingly, folliculo-stellate cells in the adenohypophysis were strongly labeled and they are known as scavengers with high lysosomal activity [34]. In the ovarian follicle, granulosa and theca cells were labeled almost uniformly probably reflecting a synchrony in cell development necessary to insure active follicular development. In contrast, heterogeneity of labeling was observed in other tissues for similar and adjacent cell types. This was particularly striking in tissues such as the epithelial cells lining the oviduct, the uterus, the epididymis and the seminal gland. In these tissues, some epithelial cells did not stain while adjacent cells were strongly labeled. This could reflect a different physiological state between these epithelial cells within the tissue.

The results observed for the bovine LAPTM4B mRNA and protein regulation, which is predominant in DF while reduced in OF, suggest a potential role of LAPTM4B in the growth of ovarian follicles. It was proposed that human LAPTM4B promoted cell proliferation since it was overexpressed in liver tumors [11], and was differentially expressed with potential tumorigenic significance associated with pituitary tumorigenesis [14]. Moreover, knockdown and overexpression studies of LAPTM4B demonstrated its capacity in binding and sequestring chemotherapeutic agents towards lysosomes thereby inhibiting their action and conferring chemotherapeutic resistance [15,35-37]. Comparatively, another member of the LAPTM superfamily, LAPTM4A, was involved in cellular detoxification processes through transport of toxic substances in late endosomes and toward lysosomes, thereby participating in cell survival [4,33]. Thus, expression of LAPTM4B in follicular and luteal cells could act as a survival factor through sequestration and detoxification of metabolites into lysosomes. Since granulosa cells of growing dominant follicle are metabolically and steroidogenically very active, LAPTM4B may confer protection against accumulation of metabolic byproducts and induction of apoptosis.
